# No Acute Effects of Cannabidiol on the Sleep-Wake Cycle of Healthy Subjects: A Randomized, Double-Blind, Placebo-Controlled, Crossover Study

**DOI:** 10.3389/fphar.2018.00315

**Published:** 2018-04-05

**Authors:** Ila M. P. Linares, Francisco S. Guimaraes, Alan Eckeli, Ana C. S. Crippa, Antonio W. Zuardi, Jose D. S. Souza, Jaime E. Hallak, José A. S. Crippa

**Affiliations:** ^1^Department of Neurosciences and Behavioral Sciences, Ribeirão Preto Medical School, University of São Paulo, São Paulo, Brazil; ^2^Instituto Nacional de Ciência e Tecnologia Translacional em Medicina, Conselho Nacional de Desenvolvimento Científico e Tecnológico, Brasília, Brazil; ^3^Department of Pharmacology, Ribeirão Preto Medical School, University of São Paulo, São Paulo, Brazil; ^4^Department of Pediatrics, Neuropediatrics, Federal University of Paraná, Curitiba, Brazil

**Keywords:** cannabidiol, CBD, polysomnography, sleep-wake cycle, sleep

## Abstract

Cannabidiol (CBD) is a component of *Cannabis sativa* that has a broad spectrum of potential therapeutic effects in neuropsychiatric and other disorders. However, few studies have investigated the possible interference of CBD on the sleep-wake cycle. The aim of the present study was to evaluate the effect of a clinically anxiolytic dose of CBD on the sleep-wake cycle of healthy subjects in a crossover, double-blind design. Twenty-seven healthy volunteers that fulfilled the eligibility criteria were selected and allocated to receive either CBD (300 mg) or placebo in the first night in a double-blind randomized design (one volunteer withdrew from the study). In the second night, the same procedure was performed using the substance that had not been administered in the previous occasion. CBD or placebo were administered 30 min before the start of polysomnography recordings that lasted 8 h. Cognitive and subjective measures were performed immediately after polysomnography to assess possible residual effects of CBD. The drug did not induce any significant effect (*p* > 0.05). Different from anxiolytic and antidepressant drugs such as benzodiazepines and selective serotonin reuptake inhibitors, acute administration of an anxiolytic dose of CBD does not seem to interfere with the sleep cycle of healthy volunteers. The present findings support the proposal that CBD do not alter normal sleep architecture. Future studies should address the effects of CBD on the sleep-wake cycle of patient populations as well as in clinical trials with larger samples and chronic use of different doses of CBD. Such studies are desirable and opportune.

## Introduction

Cannabidiol (CBD), one of the major compounds of *Cannabis sativa*, has been shown to have several therapeutic effects including antipsychotic (Zuardi et al., 1991; Leweke et al., 2000; Moreira et al., 2006), antidepressant (Zanelati et al., 2010), anti-epileptic (Devinsky et al., 2016) anti-inflammatory (Esposito et al., 2013), and analgesic properties (Boychuk et al., 2015), besides improving Parkinson’s disease symptoms (Chagas et al., 2014c).

Cannabidiol may play a therapeutic role in sleep regulation (Monti, 1977; Chagas et al., 2014b). In healthy volunteers with regular sleep cycle, 600 mg of CBD induced sedative effects (Zuardi et al., 1993), whereas in subjects with insomnia, acute use of CBD (160 mg/day) was associated with an increase in total sleep time and less frequent awakenings (Carlini and Cunha, 1981). Daily CBD doses of 40, 80, or 160 mg were shown to reduce dream recall and did not cause ‘hangover’ effects compared to placebo (Carlini and Cunha, 1981).

At lower doses, CDB (15 mg/day) co-administered with tetrahydrocannabinol (THC, 15 mg/day) increased wakefulness (Nicholson et al., 2004). More recently, Chagas et al. (2014b) investigated the effects of chronically administered CBD (75–300 mg per day for 6 weeks) in patients with Parkinson’s disease and found a reduction in symptoms of REM sleep behavior disorder. After discontinuation of the drug, the frequency of symptoms returned to baseline levels, prior to treatment with CBD. Finally, CBD-enriched extract was described as a safe treatment for reducing anxiety and improving sleep in a young girl with post-traumatic stress disorder (Shannon and Opila-Lehman, 2016).

Although some studies have demonstrated the potential effect of CBD on sleep behavior, research about the effects of CBD on the slow wave sleep (SWS) of humans with regular sleep is still lacking. The impact of CBD on sleep, possible side-effects or the advantages of lack of them, including objective measures through polysomnography, has not yet been investigated. Thus, the objective of the present study was to assess the effect of the acute administration of an anxiolytic dose (300 mg, Zuardi et al., 1993, 2017) of CBD on sleep in healthy volunteers by means of cognitive and subjective measures and polysomnography exams.

## Materials and Methods

### Screening Procedure and Clinical Assessment

**FIGURE 1:**
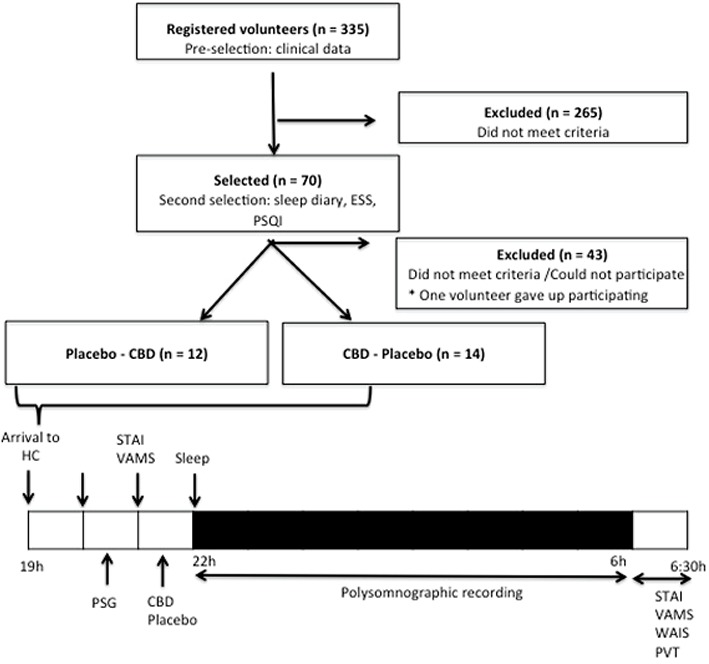
Schematic representation of the participants selection and of the protocol – this was a four period crossover study. CBD, cannabidiol; ESS, Epworth Sleepiness Scale; PSQI, Pittsburgh Sleep Quality Index; PSG, polysomnography; PVT, Psychomotor Vigilance Test; STAI, State-Trait Anxiety Inventory; TCLE, written informed consent form; VAMS, Visual Analog Mood Scale; WAIS, Wechsler Adult Intelligence Scale.

Participants were recruited through advertisements in the local media of the city of Ribeirão Preto, São Paulo, Brazil. Initially, 335 individuals who were interested in participating were evaluated, 265 of whom were excluded in the recruitment interview (which contained questions about clinical data, demographics, psychiatric symptoms, sleep patterns, among others). The remaining 70 participants were asked to keep a sleep log and completed the rating scales on sleep patterns (ESS, Epworth Sleepiness Scale*;* PSQI, Pittsburgh Sleep Quality Index). After these procedures, 27 participants were considered eligible for the study (**Figure 1**) and were randomized into two groups (group 1: placebo – CBD, group 2: CBD – placebo) matched in terms of sex, age, and years of education. To ensure the adequacy of the matching procedure, one participant of each pair had his treatment blindly chosen between the two treatment options available and the other participant (matched to the first one) was assigned to the remaining option.

The exclusion criteria for the trial were: (i) presence of organic brain syndromes; (ii) use of psychoactive drugs, including nicotine; (iii) presence of general medical conditions, assessed by the patient’s report during the interview and/or through physical examination; (iv) presence of psychiatric disorders (assessed with the SCID-IV); (v) pregnancy; (vi) previous history of any sleep disorder (based on the Pittsburgh Sleep Quality Index - PSQI); and (vii) recent changes in sleep time (variation of more than 2 h in the last 7 days, measured through the sleep log). Thus, the volunteers were all non-smokers and had not taken any medications for at least 3 months before the study. Moreover, none of them had used marijuana more than five times in their lives (no use in the last year) and none had ever used any other illegal drug. All subjects gave their written consent to participate after being fully informed about the research procedures, which were approved by the Hospital das Clínicas de Ribeirão Preto of University of São Paulo ethics committee (HCRP No. 17912/2013).

### Instruments

The following instruments were used: (a) Visual Analog Mood Scale – VAMS (Norris, 1971); (b) State-Trait Anxiety Inventory – STAI (Spielberger et al., 1970), translated and adapted to Brazilian Portuguese by Gorenstein and Andrade (1996); (c) Epworth Sleepiness Scale – ESS (Johns, 1991); (d) Pittsburgh Sleep Quality Index – PSQI (Buysse et al., 1989); (e) digit symbol substitution and symbol copying tests of the Wechsler (1955) Adult Intelligence Scale – WAIS; and (f) Psychomotor Vigilance Test – PVT (Graw et al., 2004; as made available by the National Center on Sleep Disorders Research).

### Polysomnography

The apparatus used for the polysomnography exams consisted of different devices including electroencephalogram with the international 10–20 system (to rule out the occurrence of epileptic seizures), electrooculogram, electromyogram of chin muscles and upper and lower limbs, nasal pressure cannula, oral thermistor, thoracic and abdominal respiratory inductive plethysmography straps, pulse oximetry, electrocardiogram, and snoring and body position sensors. Video and sound were also recorded during the exam.

Polysomnography recordings were obtained through a computerized system (BrainNet BNT; *LYNX Tecnologia Eletrônica*, Rio de Janeiro, Brazil). Sleep stages were recorded in periods of 30 s, according to the criteria established by Rechtschaffen and Kales (1968). The following polysomnographic parameters were evaluated: total sleep time (TST, min), sleep onset latency (min), rapid eye movement (REM) onset latency (min), wake after sleep onset (min), wake after sleep onset index (h), apnea index (h), hypopnea index (h), respiratory disturbance index (RDI, h), sleep efficiency (%), stage 1 sleep (%), stage 2 sleep (%), stage 3 sleep (%), REM (%), lowest saturation (%), and baseline saturation (%).

### Drugs

Cannabidiol (300 mg), 99.9% purity without THC (kindly supplied by STI-Pharm, Brentwood, United Kingdom) was dissolved in corn oil (Zuardi et al., 1993, 2017; Crippa et al., 2004). The same amount of corn oil was used as placebo. The drug and placebo were packed in identical gelatin capsules. The 300 mg dose was chosen based on previous studies that detected the acute anxiolytic effect of this dose (Zuardi et al., 1993, 2017) and the studies by Chagas et al. (2014b) and Chagas et al. (2014c), in which this dose caused a reduction in the frequency of REM sleep behavioral events and improving quality of life (including sleep) in patients with Parkinson’s disease, respectively. The time of drug delivery was based on previous studies that showed that the peak plasma concentration of an oral dose of CBD normally occurs 1–2 h after ingestion (Agurell et al., 1981; Crippa et al., 2004, 2010; Borgwardt et al., 2008; Fusar-Poli et al., 2009; Zuardi et al., 2017).

### Procedure

Subjects were instructed to abstain from alcohol for 24 h and caffeine for at least 24 h before each visit to the laboratory. Subjects who reported having less than 6 h of sleep the previous night were excluded from the trial. After at least 8 h of fasting, subjects were instructed to have a light, standardized meal 2 h before the experiment. For the present study, a randomized, double blind, and crossover model was used. Once one volunteer gave up participating the study, the 26 participants were assessed on two different occasions, in a 2-week interval, with identical procedures except for the substance that was administered. In each visit, participants were first submitted to a cognitive and subjective evaluation, then an oral dose of CBD (300 mg) or placebo was administered 30 min before the polysomnographic recordings began.

After arrival at the Clinical Research Unit of the Ribeirão Preto Medical School University Hospital (Ribeirão Preto, Brazil) written informed consent was signed, the subjective measures (STAI, VAMS) of the participants were collected and the electrodes used for the polysomnography exam were placed. Next, the subjective measures were completed once again (STAI, VAMS) and, 30 min before the beginning of the polysomnographic examination, the single dose of CBD (300 mg) or placebo was administered. Polysomnography recordings were performed over 8 h. On the morning after the examination, the electrodes were removed from the subject and the VAMS, STAI, WAIS, and PVT were completed. The steps of the experimental protocol are shown in **Figure 1**.

### Statistical Analysis

Clinical and demographic data were analyzed with descriptive statistics and expressed in terms of mean ± standard error of the mean. The Kolmogorov-Smirnov test was used to check for normality. Non-parametric Wilcoxon or Friedman tests analyzed results that failed this test. The remained data was analyzed by two-way repeated-measures ANOVA. A preliminary analysis indicated no gender effect; thus, the factors analyzed were drug, order of drug administration (placebo-CBD versus CBD-placebo), and the interaction between drug and phase. A three-way repeated-measures ANOVA was employed to analyze data throughout the three phases of each exam. In case of significant interactions, paired Student’s *t*-tests were performed at each phase and/or order to compare the differences between groups. In case of significant time effect, the Bonferroni’s *post hoc* test was used for multiple comparisons. In cases where sphericity conditions were not reached, the degrees of freedom of the repeated factor were corrected with the Huynh-Feldt epsilon. All the analyses were performed with the Statistical Package for the Social Sciences (SPSS) v.20.0.

## Results

The demographic characteristics of the subjects as well as measures from the scales assessing sleep and body mass index are described in **Table 1**.

**Table 1:** Demographic characteristics.

Demographic characteristics	*N* = 26
**Gender**	
Male	12
Female	14
Age (years)	29.3 (8.5)
Education (years)	14.43 (0.6)
ESS	7.3 (3.2)
BMI	24.6 (3.1)

*Data are expressed as mean ± sem (ESS = Epworth Sleepiness Scale; BMI = body mass index).*

The data of the PSQI, used in the selection of volunteers, are reported in **Table 2**. The results are presented according to the seven components assessed in the PSQI.

**Table 2:** Average scores in the seven components of the PSQI.

Component	Score (average)
1. Subjective sleep quality	1
2. Sleep latency	1
3. Sleep duration	0
4. Habitual sleep efficiency	1
5. Sleep disturbances	1
6. Use of sleeping medication	0
7. Daytime dysfunction	0
PSQI – Total^∗^	4

*^∗^Total PSQI – sum of the scores in all components.*

The data obtained in the seven components and the total PSQI score are indicative of good sleep quality. Total PSQI scores greater than five suggest difficulties in at least two components or moderate difficulties in more than three components (Buysse et al., 1989).

The main polysomnographic results of the administration of CBD and placebo are described in **Table 3**. The comparative analyses between CBD and placebo indicate that none of the parameters evaluated presented statistically significant changes.

**Table 3:** Polysomnographic parameters measured after the administration of CBD and placebo.

Parameter	Placebo	CBD	*F* value	*Z value*	*p* value
TST (min)	389.3 (66.2)	388.1 (51.4)	-	0.14	NS
Sleep onset latency (min)	13.4 (12.8)	9.8 (8.4)	-	1.32	NS
REM onset latency (min)	138.1 (72.7)	121.3 (62.2)	-	0.97	NS
Wake after sleep onset (min)	46.2 (41.8)	47.10 (41.2)	-	0.13	NS
Wake after sleep onset index (h)	18.62 (8.47)	24.7 (33.4)	0.004	-	NS
Apnea index (h)	0.31 (1.3)	0.03 (0.1)	0.95	-	NS
Hypopnea index (h)	2.2 (3.7)	1.8 (1.7)	0.32	-	NS
RDI (h)	2.8 (4.5)	2.2 (2.0)	-	0.30	NS
Sleep efficiency (%)	85.3 (13.2)	87.1 (10.2)	-	0.96	NS
Stage 1 sleep (%)	4.9 (2.1)	5.1 (3.4)	-	0.20	NS
Stage 2 sleep (%)	45.3 (8.4)	45.3 (8.4)	-	0.08	NS
Stage 3 sleep (%)	31.7 (11.1)	31.7 (11.6)	-	0.39	NS
REM (%)	17.6 (4.1)	16.6 (4.4)	0.008	-	NS
Lowest saturation (%)	84.53 (14.4)	90.03 (4.7)	-	1.09	NS
Baseline saturation (%)	96. (1.05)	96.65 (1.05)	1.805	-	NS

*CBD = cannabidiol; REM = rapid eye movement sleep; RDI = respiratory disturbance index; TST = total sleep time; I: number of events per hour. Results are expressed as means ± sem.*

No statistically significant differences were found between groups in the VAMS, STAI, Digit Symbol Substitution and Symbol Copying Tests, and PVT. In the analysis of the WAIS, the results in the Symbol Copying Tests showed no effects of drug (*F*_1,24_ = 2.46; *p* > 0.05) or order of administration (*F*_1,24_ = 0.44; *p* > 0.05), but the interaction between drug and order was significant (*F*_1,24_ = 4.9, *p* < 0.05). To check if this interaction could have potentially interfered with the results, we split the subjects, comparing the placebo and CBD groups separately in the two orders (first placebo or CBD). Again, there was no difference between groups in the two situations.

## Discussion

We found no significant differences in polysomnography results following the administration of CBD and placebo to healthy volunteers. Likewise, there were no statistically significant changes in the subjective and cognitive measures collected during the two nights of polysomnographic exams.

Several parameters were recorded during polysomnography, considering that the essential tests for sleep staging are electroencephalogram, electrooculogram, and electromyogram. Given the lack of studies on the effect of CBD on human polysomnography-monitored sleep, other parameters were selected based on studies that tested the effect of other drugs in healthy volunteers (Orr et al., 2012; Yadollahi et al., 2014). When comparing our polysomnographic data with results from other studies that used placebo in healthy volunteers, similar findings were observed (Buysse et al., 1989; Sabbatini et al., 2005; Fidan et al., 2011; Feld et al., 2013; Wilson et al., 2015).

No statistically significant changes were found between the three different time points in the four factors evaluated by the VAMS and, as well as in the STAI. These results suggest that none of the different moments of the exams were subjectively rated as anxiogenic, sedative, uncomfortable or as producing cognitive impairment. It should be noted here that, unlike other medications, the anxiolytic effect of CBD is only observed when given to subjects in obviously anxiogenic situations (Zuardi et al., 1993, 2017; Bergamaschi et al., 2011a; Crippa et al., 2010).

In the present study, we found no residual effects of CBD on cognitive or psychomotor functions compared to placebo, as measured by the Digit Symbol Substitution and Symbol Copying subscales of the WAIS, which have been described as sensitive measures of residual drug effects (Garber et al., 2004; Rosenberg et al., 2007).

Although no previous study on sleep and CBD applied these specific measures, our findings are consistent with a study on multiple sclerosis that used the digits test to assess possible changes in disease status following the administration of CBD associated with THC, in which no significant change was recorded (Vaney et al., 2004).

It is known that lack of sleep can interfere with certain aspects of cognitive functioning, such as attentional levels (Goel et al., 2009) and PVT, which has a high sensitivity to measure responses that require selective attention (Basner and Dinges, 2011). However, the results of the present study did not show any significant impairment in either the reaction time or number of errors measured by the PVT, suggesting that the attention levels of the volunteers were preserved in the morning after the sleep assessment, regardless of the administration of CBD or placebo. Not having administered the PVT test before CBD and placebo administration does not significantly affect the conclusions once the study does not intend to assess the effect of CBD on baseline vigilance (which would require comparison with baseline PVT results), but to rather evaluate if CBD may be safely administered to patients without affecting their vigilance state overall, such that the patients may safely conduct every-day tasks, like for example driving.

Earlier preclinical studies have suggested that the therapeutic effects of CBD might depend on the presence of specific clinical conditions. As an example, Campos et al. (2013) showed that the chronic use of CBD for 2 weeks, while not directly increasing hippocampal neurogenesis, prevented its decrease by unpredictable chronic stress. Thus, the absence of changes in the sleep of healthy volunteers treated with CDB in our study should not be considered as a final indication that CBD could not have positive effects in patients with sleep disorders.

It is known that a major problem of several medications used in the treatment of clinical anxiety and depression is their effect on sleep architecture. Benzodiazepines are an example, since despite the rapid onset of their anxiolytic action, these drugs may produce undesirable side effects such as the increase in non-REM stage 2 sleep and reduction of SWS (Borbély et al., 1985). Long-term use of benzodiazepines may also cause reduction of SWS, loss of efficacy in the treatment of insomnia, alterations in electroencephalogram results during sleep (Poyares et al., 2004) and cognitive dysfunction, even after drug discontinuation (Stewart, 2005).

Likewise, selective serotonin reuptake inhibitors (SSRIs) and selective serotonin and norepinephrine reuptake inhibitors (SNRIs) may interfere with sleep architecture and decrease restorative sleep, leading to increased awakenings, reduced REM sleep, increased REM latency, as well as increased periodic limb movement during sleep (Feige et al., 2002). In addition, SSRIs and SNRIs have been associated with REM sleep without atonia, characterized by increased tonic or phasic motor activity in electromyographic channels during REM sleep (Schenck et al., 1992; American Academy of Sleep Medicine, 2014; Lee et al., 2016).

Cannabidiol’s anti-anxiety (Zuardi et al., 1993, 2017; Crippa et al., 2009; Bergamaschi et al., 2011b) and antidepressant (Saito et al., 2010; Zanelati et al., 2010) potential seems to differ from other drugs with effects on the central nervous system, since we found no alterations in sleep architecture. Additionally, studies on the anxiolytic, antipsychotic and antiparkinson effects of CBD described no sedation or drowsiness side effects in their volunteers (Zuardi et al., 1993; Crippa et al., 2004; Fusar-Poli et al., 2009; Chagas et al., 2014a). These findings complement the literature on the few significant side effects resulting from the administration of CBD to humans in a wide range of doses, administered chronically or acutely (Bergamaschi et al., 2011b; Kerstin and Grotenhermen, 2017). It seems, therefore, that CBD has an adequate safety profile with good tolerability and does not affect psychomotricity or cognition (Hayakawa et al., 2007; Crippa et al., 2010; Bergamaschi et al., 2011b; Kerstin and Grotenhermen, 2017). This is particularly important in Parkinson’s disease, where motor and cognitive symptoms play a central role.

The relative representativeness of the small sample size and the use of a single dose of CBD can perhaps be regarded as a limitation of our study, as it does not allow the assessment of the effects of chronic treatment with CBD on sleep. In the study by Chagas et al. (2014b), for example, CBD was chronically administered for 6 weeks to patients with Parkinson’s disease and REM sleep behavior disorder. Since the effects of CBD are biphasic (Zuardi et al., 2017), the use of a single dose also limits the interpretation of the present findings. Moreover, monitoring changes in sleep using a conventional polysomnography presents some intrinsic limitations, as it is insufficient alone to detect drug-induced changes of the sleep EEG. For this purpose, a spectral analysis or a similar procedure is also needed. Conversely, the use of preclinical polysomnography to characterize drug-induced sleep disturbances has been increasingly recommended in the regulatory context (Authier et al., 2016). Finally, it is essential to evaluate the effects of CBD in a larger sample and in individuals diagnosed with sleep disorders in addition to healthy volunteers.

Despite these limitations, this is the first controlled study to evaluate the effects of CBD on sleep architecture using polysomnography. Although the absence of interference with the sleep cycle is not sufficient for concluding that sleep is not affected, the results obtained contribute for the understanding of the effects of CBD in the modulation of sleep in humans.

## Conclusion

We found no differences between CBD and placebo in respect to polysomnographic findings or cognitive and subjective measures in a sample of healthy subjects. Unlike widely used anxiolytic and antidepressant drugs such as benzodiazepines and SSRIs, the acute administration of an anxiolytic dose of CBD does not appear to interfere with the sleep cycle of healthy volunteers. Future studies should address the effects of CBD on the sleep-wake cycle of patient populations as well as evaluate the chronic effects of CBD in larger samples of patients with sleep and neuropsychiatric disorders.

## Author Contributions

JC, JH, FG, AZ, and AE: conception or design of the work. IL, JS, and AE: data collection. AE, FG, IL, AZ, and AC: data analysis and interpretation. JC, IL, AZ, JH, and FG: drafting the article. JC, JH, FG, AZ, AE, AC, JS, and IL: critical revision of the article. JC, JH, FG, AZ, AE, AC, JS, and IL: final approval of the version to be published.

## Conflict of Interest Statement

AZ, JH, FG, and JC are co-inventors (Mechoulam R, JC, FG, AZ, JH, and Breuer A) of the patent “Fluorinated CBD compounds, compositions and uses thereof. Pub. No.: WO/2014/108899. International Application No.: PCT/IL2014/050023” Def. US no. Reg. 62193296; 29/07/2015; INPI on 19/08/2015 (BR1120150164927). The University of São Paulo has licensed the patent to *Phytecs Pharm* (USP Resolution No. 15.1.130002.1.1). The University of São Paulo has an agreement with *Prati-Donaduzzi* (Toledo, Brazil) to “develop a pharmaceutical product containing synthetic cannabidiol and prove its safety and therapeutic efficacy in the treatment of epilepsy, schizophrenia, Parkinson’s disease, and anxiety disorders.” JH and JC have received travel support from and are medical advisors of BSPG-Pharm. AZ is medical advisor of BSPG-Pharm. The other authors declare that the research was conducted in the absence of any commercial or financial relationships that could be construed as a potential conflict of interest.
